# Where Are the Women? Descriptive Representation and COVID-19 in U.K. Daily Press Briefings

**DOI:** 10.1017/S1743923X20000513

**Published:** 2020-07-30

**Authors:** Jessica C. Smith

**Affiliations:** University of Southampton

**Keywords:** Descriptive representation, substantive representation, COVID-19, British politics

## Abstract

As governments tackle the ongoing COVID-19 pandemic, both the role of women in governments and the specific effect of the pandemic on women have come under scrutiny. This research note examines the descriptive representation of women in the U.K. government's response to the coronavirus. It finds that 43% of the government's daily press briefings featured an all-male lineup with no female politician or expert present. In particular, female politicians are missing, with only one female cabinet member ever leading the briefing. Women's (in)visibility raises concerns about the legitimacy of democratic decisions and likely has policy consequences: women's absence may exacerbate gendered inequalities resulting from the crisis.

When Donald Trump announced the first iteration of his White House Coronavirus Task Force, he faced immediate criticism that all 12 members were men. The U.S. case is not unique; women make up only 20% of the World Health Organization's Emergency Committee on COVID-19.^1^ Yet the coronavirus has explicitly gendered effects. We know from previous epidemics that ignoring structural gender inequalities in crisis response can further reinforce or exacerbate these inequalities (Davies and Bennett 2016). The presence of women in government responses to the COVID-19 pandemic may mitigate the detrimental gendered effects, given that the presence of women is more likely to lead to attention to women's interests. This research note examines the descriptive representation of women in the U.K. response to the coronavirus. It finds that 42.5% of the government's daily briefings featured an all-male lineup with no female politician or expert present. This has normative and symbolic consequences for the legitimacy of the government response and possible policy consequences for the current and future gendered impact of COVID-19.

## DESCRIPTIVE AND SUBSTANTIVE REPRESENTATION

Descriptive representation is “the making present of something absent by resemblance or reflection, as in mirror or art” (Pitkin 1967, 11)—that is, the extent to which elected bodies represent the population in terms of demographics and other characteristics. There are normative grounds for ensuring gender balance in descriptive representation. Democratic theorists contend that the systematic exclusion of certain groups from decision-making means that legislative outcomes, processes, and institutions are not legitimate (Mansbridge 1999; Phillips 1998). Such inequality in representation is a concern for the health of a democracy. Citizens may reflect these normative concerns; experimental evidence suggests that voters’ perceived procedural legitimacy of a government decision increases when there is a gender balance among those deciding the outcome (Clayton, O'Brien, and Piscopo 2019).

Second, there is a symbolic argument for descriptive representation, as including those who were previously excluded from institutions symbolizes their equality (Phillips 1998). Furthermore, it is important for citizens to “see” themselves represented. Greater female representation can increase women's political knowledge (Dassonneville and McAllister 2018), discussion of politics among younger women (Wolbrecht and Campbell 2017), and girls’ reported anticipated political involvement (Campbell and Wolbrecht 2006).

Representation also has policy consequences. Feminist scholars link descriptive representation to substantive representation (the actions of the representatives). “There are particular needs, interests, and concerns that arise from women's experience, and these will be inadequately addressed in a politics that is dominated by men” (Phillips 1998, 66). There is mixed evidence for the link between women's presence and substantive representation of interests or issues (e.g., Homola 2019). We know that this link is imperfect as representation varies dependent on many factors, such as context, institutions, and the diversity of female representatives (Childs and Lovenduski 2013). However, on balance, when women are present in decision-making bodies, the consequence is better outcomes for women. Women representatives identify their sex as a constituency (Childs 2004) and are more likely to act for women than men (Annesley et al. 2014; Catalano 2009; Celis 2013; Taylor-Robinson and Heath 2003; Wängnerud 2009).

In this note, the sex of both government ministers and expert advisers who attend the daily briefings is observed. Work on representation began with the legislative arena but has expanded to institutions beyond legislatures, including executives, agencies, parties, and social organizations (Annesley, Gains, and Franceschet 2019; Bergqvist 1999; Breitenbach 1981; McBride and Mazur 2010; Murray 2008). As Saward (2010) argues, representation can occur in multiple sites, beyond legislatures, and even outside of institutions and formal politics. These briefings are the public face of the government's decision-making on COVID-19 and therefore have clear symbolic consequences for female representation. Furthermore, although the briefings do not explicitly identify those who are responsible for policy, they likely reflect the priorities in the government's decision-making processes.

Considering women's descriptive representation in the coronavirus pandemic is especially important given the pandemic's gendered effects. Major health crises, such as the Ebola and Zika epidemics, have been shown to exacerbate gendered inequalities, in both cases “leaving structural gender inequalities out of the crisis response has further compounded those [gender] inequalities” (Davies and Bennett 2016, 1044). In the case of COVID-19, there are a myriad of gendered inequalities that could be aggravated, which need to be recognized in policy responses. As people work from home with schools closed, the inequitable division of domestic labor and child care may negatively impact women. Initial evidence finds that U.K. mothers are more likely to have quit or lost their job since the beginning of the lockdown, and they are spending less time on paid work and more on household responsibilities than fathers (Andrew et al. 2020). Survey evidence from the United States, United Kingdom, and Germany finds that in the United States and United Kingdom, women are significantly more likely to have lost their jobs due to COVID-19 (Adams-Prassi et al. 2020). Instances of domestic violence have seen significant increases in lockdown (Williamson, Lombard, and Brooks-Hay 2020). Moreover, women make up 70% of health and social care workers on the front lines of the pandemic.^2^ These gendered impacts have policy implications as the government negotiates lockdown and the subsequent economic impact of COVID-19.

As discussed, women's presence in decision-making means that women's interests are more likely to be addressed in policy discussions and outcomes. Policies addressing the gendered effects of COVID-19 include increasing eligibility for and the rate of statutory sick pay, increasing rates of child benefit and child care allowance, and strengthening out-of-work support (Resolution Foundation 2020; WBG 2020).

## METHODOLOGY

Since March 16, 2020, the U.K. government has conducted daily press briefings on the coronavirus, broadcast on national television. A cabinet member or the prime minister leads the briefing, accompanied by one or two experts, usually scientific advisers. The sex of all ministers (including the prime minister) and scientific or other advisers who conducted the briefings was collected. Since the pandemic, and the daily briefings, were ongoing at the time of writing, data are included from the first briefing (March 16, 2020) until the beginning of the easing of some lockdown measures (May 10, 2020). The sex of politicians and experts was hand coded by the researcher by viewing the daily briefings.^3^

## RESULTS

### Sex of Politicians

**Figure 1.**
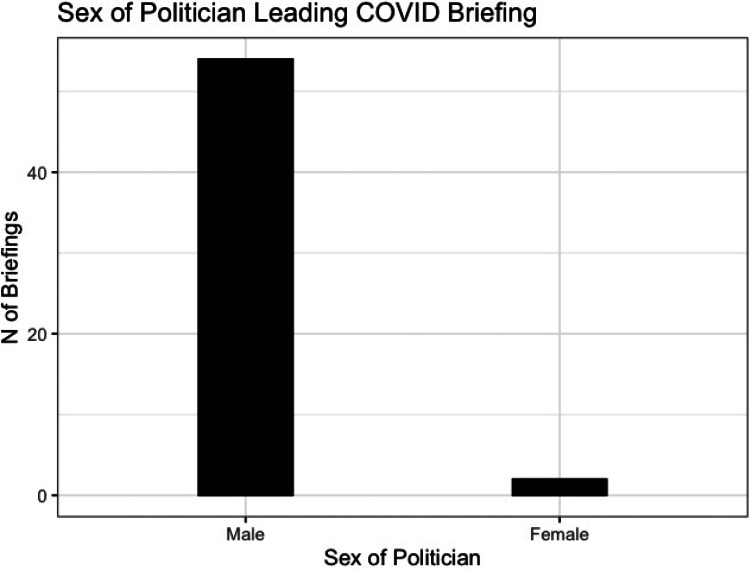
Sex of lead politician.

**Figure 2.**
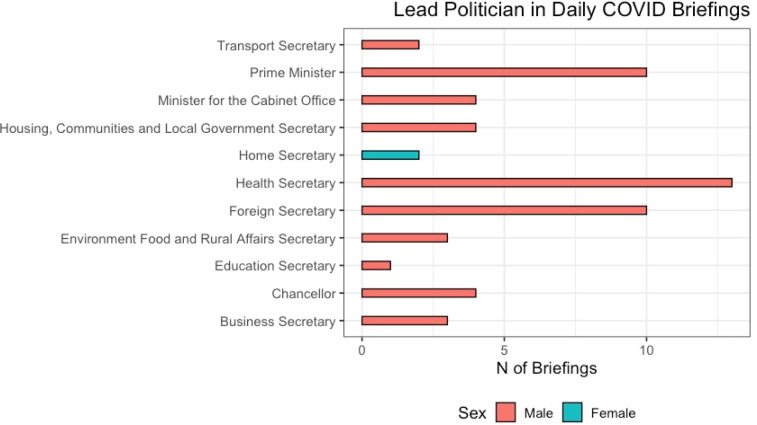
Position and sex of lead politician

In total, 7%, only 2 of the 56 briefings, were led by a female politician, in both cases Home Secretary Priti Patel (Figure 1).^4^ This difference is not driven by the prime minister: only 10 of the briefings in this time period were led by the prime minister (see Figure 2), in part because he contracted the coronavirus and required subsequent hospitalization.

A range of (male) cabinet ministers led the daily briefings (Figure 2). The prime minister and the health secretary were the most frequent leads alongside the foreign secretary (given the conventions of this position being deputized when the prime minister is ill), all of whom are male. Following these, a variety of cabinet members led briefings, all but one of them male. Women's underrepresentation here could be attributed to the diversity of Boris Johnson's cabinet: 6 of its 21 members are women. Yet only 17% (1 out of 6) women ministers led a briefing, compared with two-thirds (10 out of 15) of male cabinet ministers. Many of the women occupy cabinet positions that relate to COVID-19: for example, the secretary of state for work and pensions and the secretary of state for international trade (who is also minister for women and inequalities).

### Sex of Experts

**Figure 3.**
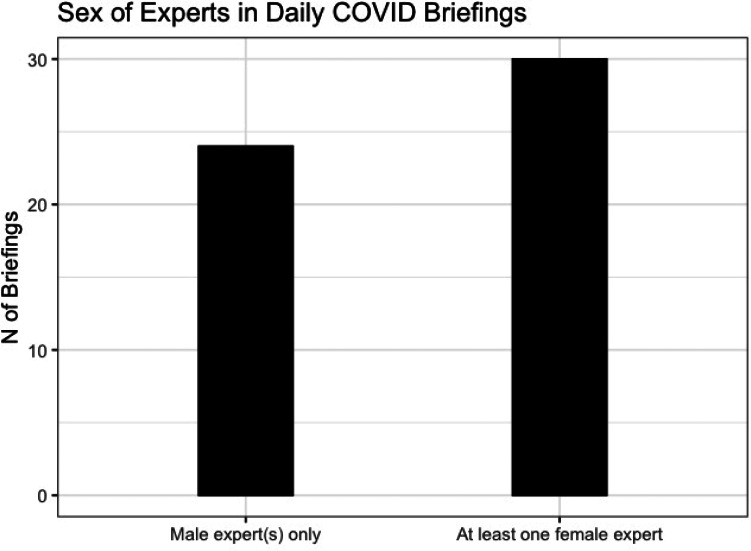
Sex of experts in daily briefings.

**Figure 4.**
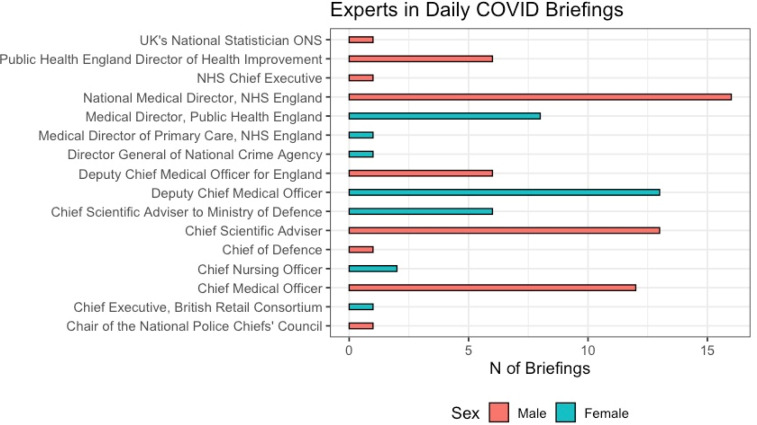
Position and sex of experts.

In each briefing save two, which were statements made by the prime minister only, a politician was joined by at least one expert, usually two. Most often, these were health or scientific advisers (Figure 4). Figure 3 shows that in total, 44.4% of the briefings did not include a female expert (24 out of 54).

Figure 4 shows that female experts were, however, more visible than female politicians: 44% (7 out of 16) of the advisers who attended daily briefings were women, and one of the three experts who appeared most frequently was a woman.

### The Full Lineup

**Figure 5.**
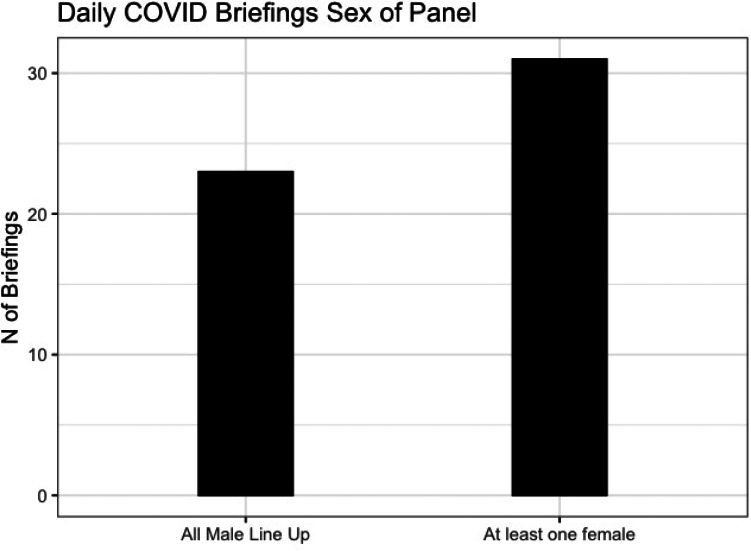
Number of briefings that included a female expert or politician.

Given the underrepresentation of women among both politicians and, to a lesser extent, experts, the U.K. public was often presented with an all-male panel at the daily briefings. Figure 5 shows that 43% of briefings (23 out of 54)^5^ featured an all-male lineup—that is, no female politician or expert was included.

## DISCUSSION

The U.K. government presented a very male face in its public response to the coronavirus pandemic. Female politicians were essentially absent from its daily briefings, with only 2 out of 56 briefings led by a female cabinet member. The picture was slightly better for the experts who attended the daily briefings. The female deputy chief medical officer was the joint-second most frequent adviser who attend briefings. Yet 44% (24 out of 54) of the briefings did not include a female expert, and 43% (23 out of 54) featured an all-male lineup with no female politician or expert present.

Although this is only a single-country case, these results are important from a normative, symbolic, and policy perspective. Women's (in)visibility raises concerns about the legitimacy of democratic decisions and likely has policy consequences: women's absence may exacerbate gendered inequalities resulting from the crisis. Symbolically, women are not “seeing” themselves represented, and this has potential consequences for female political engagement. The United Kingdom's press briefings are another instance of the underrepresentation of women in frontline politics and an instance that could have severe and long-lasting effects.

## Footnotes

1. Women in Global Health, “Operation 50/50: Women's Perspectives Save Lives,” https://www.womeningh.org/operation-50-50 (accessed May 19, 2020).

2. Women in Global Health, “Operation 50/50.”

3. Sex was measured as an observed characteristic. This coding is reliable, as I am unaware of any self-identification statement by the relevant politicians or experts that would contradict the researcher's coding.

4. In three briefings, a cabinet minister joined the prime minister alongside an expert. It was coded according to which politician led the briefing in terms of opening the briefing and taking questions.

5. Excludes two briefings at which the prime minister made a statement on his own.
